# Characterization of Early Microbial Communities on Volcanic Deposits along a Vegetation Gradient on the Island of Miyake, Japan

**DOI:** 10.1264/jsme2.ME13142

**Published:** 2014-01-24

**Authors:** Yong Guo, Reiko Fujimura, Yoshinori Sato, Wataru Suda, Seok-won Kim, Kenshiro Oshima, Masahira Hattori, Takashi Kamijo, Kazuhiko Narisawa, Hiroyuki Ohta

**Affiliations:** 1 United Graduate School of Agricultural Science, Tokyo University of Agriculture and Technology, 3–5–8 Saiwai-cho, Fuchu-shi, Tokyo 183–8509, Japan; 2 Ibaraki University College of Agriculture, 3–21–1 Chuou, Ami-machi, Ibaraki, Japan; 3 National Research Institute for Cultural Properties, Tokyo, 13–43 Ueno Park, Taito-ku, Tokyo 110–8713, Japan; 4 Department of Computational Biology, Graduate School of Frontier Science, The University of Tokyo, 5–1–5 Kashiwanoha, Kashiwa, Chiba 277–8568, Japan; 5 Faculty of Life and Environmental Science, University of Tsukuba, 1–1–1 Tennodai, Tsukuba, Ibaraki 305–8572, Japan

**Keywords:** volcanic deposits, early microbial community, 16S rRNA gene, 18S rRNA gene, pyrosequencing, plant-microbe interaction

## Abstract

The 2000 eruption of Mount Oyama on the island of Miyake (Miyake-jima) created a unique opportunity to study the early ecosystem development on newly exposed terrestrial substrates. In this study, bacterial and fungal communities on 9- and 11-year-old volcanic deposits at poorly to fully vegetation-recovered sites in Miyake-jima, Japan, were characterized by conventional culture-based methods and pyrosequencing of 16S rRNA and 18S rRNA genes. Despite the differences in the vegetation cover, the upper volcanic deposit layer samples displayed low among-site variation for chemical properties (pH, total organic carbon, and total nitrogen) and microbial population densities (total direct count and culturable count). Statistical analyses of pyrosequencing data revealed that the microbial communities of volcanic deposit samples were phylogenetically diverse, in spite of very low-carbon environmental conditions, and their diversity was comparable to that in the lower soil layer (buried soil) samples. Comparing with the microbial communities in buried soil, the volcanic deposit communities were characterized by the presence of *Betaproteobacteria* and *Gammaproteobacteria* as the main bacterial class, *Deinococcus- Thermus* as the minor bacterial phyla, and Ascomycota as the major fungal phyla. Multivariate analysis revealed that several bacterial families and fungal classes correlated positively or negatively with plant species.

A number of studies have shown that microorganisms were the primary colonizers on newly exposed volcanic substrates such as lava, tephra, and volcanic ash and contributed to early ecosystem development on such new substrates (10, 29, 49, 54). Prior to colonization by plants, the pioneer microbes were found to play a significant role in the fixation of carbon and nitrogen from the atmosphere, resulting in the input of organic matter into the deposit (29–32, 36, 52, 53). Subsequent colonization of plants directly influences the deposit microbial community through litter input, root exudates, and dead root tissues (2, 7). Reciprocally, specific microbes can associate with plants through root-microbe symbiosis or have a negative effect on plants by microbial interfering actions (3, 8). Such plant-microbe interactions will drive primary ecosystem succession on the volcanic deposit. Recently, glacier forefront ecosystems were well characterized as a model of primary microbial succession on newly exposed substrates (5, 23, 33, 64). Concerning volcanic environments, information on such interactions is limited, but several studies on a volcanic desert and young volcanic deposits showed that the soil microbial biomass and community structure were influenced by colonizer plants (44, 60).

The island of Miyake (Miyake-jima) is a volcanic island situated on the western rim of the Pacific Ocean (34°05′ N, 139°31′ E) (Supplemental material, Fig. S1). In 2000, Mt. Oyama on the island erupted, ejecting large amounts of volcanic ash and forming a collapsed crater. About 60% of vegetation on the island was initially influenced by the heavy deposition of volcanic ash. After crater formation, large amounts of volcanic gas containing SO_2_ and H_2_S were emitted and caused widespread defoliation, particularly on the leeward side of Mt. Oyama (24). Vegetation has been gradually recovering from this damage in the foothills of the mountain and on the windward sides (north and northeastern), but not on the leeward sides (eastern and southwestern) due to volcanic gas (25). We have investigated early bacterial communities on the volcanic ash deposit at a completely unvegetated site near the crater and showed that the microbial community was dominated by autotrophic, N_2_-fixing Fe(II) oxidizers, *Acidithiobacillus ferrooxidans* and the *Leptospirillum* groups, by clone library analysis of 16S rRNA genes (10, 52).

The aim of this study was to characterize the microbial community of the Miyake-jima volcanic deposit on a windward side where vegetation recovered gradually, and to analyze how the early microbial community responds to the first colonizer plants. In this study, we established three sites along an elevational transect, representing sparsely grass-covered (site IG1), fully grass- and partially shrubcovered (site IG2), and fully grass- and shrub-covered land (site IG3). The investigation was designed to compare bacterial and fungal communities among these deposits by molecular approaches using the PCR-based pyrosequencing method as well as conventional measurements of population density, respiratory activity, and the substrate utilization profile.

## Materials and Methods

### Site description and sampling

Miyake-jima (55.5 km^2^ in area; highest point, 775 m), an active basalt volcano, belongs to the Fuji volcanic southern zone in the East Japan volcanic belt (Fig. S1). For detailed information on the island and the eruption in 2000, see the supplemental material. The volcanic ash deposit derived from the eruption in 2000 was characterized by high contents of fine sand (36–76%), strong acidity [pH (H_2_O), 3.1–4.0], and high amounts of exchangeable Ca^2+^ (33.5–115 cmolc kg^−1^) and Al^3+^ (0.8–10.2 cmolc kg^−1^) (26). Sites IG1 to IG3 (Fig. S1) were established along an elevational transect on the northwest side of Mt. Oyama (altitude: site IG1, 540 m; site IG2, 437 m; and site IG3, 380 m). The thickness of the volcanic deposit derived from the eruption in 2000 was 450, 330, and 280 mm at sites IG1, IG2, and IG3, respectively (Fig. S2).

The volcanic deposit in 2000 and the soil layer (buried soil) beneath the volcanic deposit were sampled on July 27, 2009 and September 5, 2011. After removing the surface litter layer, upper volcanic ash deposits were taken from 10–200 mm in depth at site IG1, 30–140 mm in depth at site IG2, and 50–160 mm in depth at site IG3, avoiding mixing of the root-rich layer. The buried soil 2 to 7 cm underneath the volcanic deposit layer was also taken from each site. At sampling, several core samples were taken from each layer up to a total of about 1 kg, mixed in sterile plastic bags, and immediately stored on ice. Finally, samples were divided into two portions and kept at 4°C and −20°C until bacteriological analysis and DNA extraction, respectively. Major roots and plant debris were removed from all samples prior to analysis and extraction.

### Chemical analysis

Total organic carbon (TOC) and total nitrogen (TN) were determined using a Shimadzu TOC analyzer (TOC-L) (Shimadzu, Kyoto, Japan) and a Yanaco CHN Corder type MT-6 (Yanaco Analytical Instruments, Kyoto, Japan), respectively. Slurry consisting of a 1:2.5 mass ratio of sample and deionized water was used to determine the pH value. The volumetric water content was analyzed by drying the material at 105°C overnight.

### Respiratory activity and substrate utilization profile

To measure *in vitro* respiratory activity (as CO_2_ evolution), 200 g volcanic deposit sample or 100 g buried soil sample were placed together with a portable wireless infrared CO_2_ monitor (C2D-W01TR or C2D-W02TR; UDOM, Mito, Japan) in a sealed 1100- mL volume plastic box. Carbon dioxide concentration in the box was recorded continuously at 27–30°C for 90 min and initial CO_2_ production rate was calculated. The assay was performed within several hours after sampling. ECO MicroPlate (BiOLOG, Hayward, CA, USA) was used for organic substrate utilization profiling as described previously (10). In brief, 1 g sample was suspended in 99 mL sterile water and then the suspensions were shaken on a reciprocal shaker at 220 strokes min^−1^ for 20 min. After centrifugation at 500×*g* for 10 min, 150 μL subsamples were inoculated into each well of the plate (triplicate).

### Enumeration methods and soil ergosterol quantification

Total direct microscopic counts (TDC) of bacteria were determined using ethidium bromide with fluorogenic dye as described previously (49). In brief, triplicate membrane filters were prepared and bacteria were counted in at least 50 randomly selected microscopic fields of each filter preparation. Culturable bacteria were enumerated on full-strength nutrient broth (NB) and 1:100 diluted nutrient broth (DNB) as the plating agar medium (38). Four replicates of sample dilutions were plated and incubated at 30°C for 28 days. Fungal propagules were counted on rose bengal agar medium (43) in four replicates. Ergosterol was determined as an indicator of fungal biomass by the method of vibration-assisted extraction followed by HPLC quantification (12, 62). The HPLC system (Tosoh, Tokyo, Japan) was essentially the same as described previously (62). A soil sample taken at a forest site unaffected by the 2000 eruption on Miyake-jima and two agricultural soils from the Field Science Center, Ibaraki University College of Agriculture were used as references.

### DNA extraction, PCR amplification, and tag pyrosequencing

Five grams of the volcanic deposit samples were used for DNA extraction, according to a method based on lysis with a high-salt extraction buffer (1.5 M NaCl) and extended heating of the sample suspension in the presence of sodium dodecyl sulfate, hexadecyltrimethyl ammonium bromide, and Proteinase K (10, 63). DNA extraction from the buried soil samples (0.5–1.0 g) was performed by ISOIL for Bead Beating (Nippon Gene, Tokyo, Japan) with skim milk powder (Wako, Osaka, Japan) according to the manufacturer’s instructions with minor modifications (47). DNA extraction was made in duplicate and the extracts were pooled. All pooled DNA samples were purified using AMPure XP magnetic purification beads (Beckman Coulter, Brea, CA, USA). The V1-V2 region in the 16S ribosomal RNA gene was amplified using universal primers 27Fmod and 338R (28) under thermal conditions of 2 min at 96°C, 20 cycles of 96°C for 30 s, 55°C for 45 s, and 72°C for 1 min, and a final extension of 72°C for 10 min on a 9700 PCR system (Life Technologies Japan, Tokyo, Japan), according to the protocol of Kim *et al.* (28). Another universal primer set 817F-1196R was used to analyze the fungal community (4), where the same pyrosequencing adaptors and barcode sequences for amplification of 16S rRNA gene were used. The 817F-1196R primer set has been shown to target a region of the fungal 18S ribosomal RNA gene, which is variable between major taxa and can permit phylogenetic analyses such as UniFrac (51). For the primer sequences, see the supplemental material. PCR was performed in the same condition with PCR amplification of 16S rRNA gene described above, except that the annealing temperature was set to 56°C. PCR products of 16S rRNA gene and 18S rRNA gene were confirmed by electrophoresis on 2% agarose gels, purified by Beckman AMPure XP magnetic purification beads, and quantified using the Quant-iT PicoGreen dsDNA Assay Kit (Life Technologies). A composite sample was prepared by pooling approximately equal amounts of PCR amplicons from each sample and subjected to pyrosequencing using the 454 GS FLX Titanium or 454 GS JUNIOR (Roche Applied Science, Penzberg, Germany) according to the manufacturer’s instructions.

### Sequence data processing and analysis

All the raw sequence data obtained from 454 pyrosequencing were assigned to each sample on the basis of their barcode sequence. Reads with an average quality value <25 and not having both universal primer sequences were filtered off. The selected reads were denoised using the ‘pre.cluster’ command in Mothur (20). PCR chimeras were filtered off using Chimera Slayer (15). To remove the small portions of unexpected archaeal sequences, the sequences of 16S rRNA genes were identified by the RDP Classifier (58), and the archaeal sequences filtered out. Further, to remove the portions of unexpected non-fungal sequences, the effective sequences of 18S rRNA gene were aligned with the SILVA small subunit ribosomal RNA (SSU rRNA) database by the basic local alignment search tool (BLAST) (50). The non-fungal sequences were filtered out. After the above operation, the sequences of each sample were defined as qualified reads and each data set was rarefied to the smallest libraries using Daisy-Chopper (available at http://www.genomics.ceh.ac.uk/GeneSwytch). To define operational taxonomic units (OTUs), pairwise distances between sequences of the trimmed data sets were calculated as the average neighbor algorithm (55). Here, OTUs were defined at an average intra-OTU sequence identity of 97%, which is the narrowest clustering distance recommended for 454 pyrosequences (35). Good’s coverage, abundance-based coverage estimator (ACE), Shannon-Wiener index (*H*′) and the inverse Simpson index (1/*D*) were calculated at the 0.03 cutoff level. Pairwise dissimilarities between samples were calculated by the weighted UniFrac metric based on a relaxed neighbor-joining tree that was built with the representative sequence for each OTU using the ‘clearcut’ command (9). The SILVA bacterial and eukaryotic trees provided by Mothur (available at http://www.mothur.org/wiki/Silva_reference_files) were used for the reference trees of UniFrac analyses. For classification, the sequences were compared to the SILVA SSU rRNA database using the Bayesian classifier and a confidence threshold of 80% (bootstrap) (34).

### Statistical analysis

Multivariate analysis of variance (MANOVA) was used to test for significant differences in the chemical and biological properties of samples and Tukey’s honestly significant difference (HSD) test was performed to determine the rank order. Significance was defined at *P* <0.05. The influence of the sampling site, sample type, and time factor on the microbial diversity indices (OTUs, abundance-based coverage estimator, Shannon index, and inverse Simpson index) was also evaluated using Student’s *t*-test and MANOVA at 0.03 cutoff with 95% confidence intervals on the R platform (available at http://www.r-project.org). Similarities and differences in bacterial and fungal community structure among the samples were examined using the weighted UniFrac distance with the principal coordinate analysis (PCoA) ordination technique. Heat maps of the most abundant 50 bacterial OTUs and 30 fungal OTUs in each sample were used to compare the major compositions of the libraries. The heat maps were constructed using the function heatmap.2 from the R package gplots (available at http://cran.r-project.org/web/packages/gplots/index.html). For better visualization of heat maps, OTUs tables were log_2_-transformed (39). Hierarchical clustering of rows and columns in the heat maps was based on Bray-Curtis similarities and used for group-average linkage. Canonical correspondence analysis (CCA) was employed to explore the relationship between microbial communities and environmental variables. The percentage abundance of bacterial families and fungal classes in each VD and S library were used as the species input, and the vegetation properties (Braun-Blanquet cover-abundance and count of plant species, and vegetation coverage) and chemical properties (pH, TOC, TN, and water content) served as the environmental input. Ordination plots of the results from CCA were performed using the function cca from the R package vegan (available at http://cran.r-project.org/web/packages/vegan/index.html).

### Sequence data accession number

The pyrosequencing reads were deposited in the DDBJ Sequence Read Archive database under accession number DRA001160.

## Results

### Vegetation characteristics

The percentages of vegetation cover at the study sites are summarized in Table 1 and the detailed vegetation profiles are shown in Fig. S3. From analysis using satellite data, the study sites were completely (site IG1) or partially (sites IG2 and IG3) unvegetated in November, 2000 (59). In 2009, the deposit at site IG1 supported very limited growth of low grass species (<1 m in height), *Miscanthus condensatus* and *Calamagrostis autumnalis*. The percent coverage of low grass species was 20% in 2009 and increased to 45% in 2011, accompanied with the growth of other low grass species, *Polygonum cuspidatum* var. *terminale*, *Carex oshimensis*, and *Carex okuboi*. High grass species (>1 m in height) and shrub species did not occur on the deposit at the site. Sites IG2 and IG3 were characterized by the vigorous growth of *M. condensatus* up to 2 m in height with high coverage (90 to 100%). A deciduous broad-leaved tree, *Alnus sieboldiana*, was established at the sites after the eruption and its coverage at site IG2 was 15% and 25% in 2009 and 2011, respectively, and that at site IG3 was 65% and 60% in 2009 and 2011, respectively.

### Chemical and biochemical characteristics

Chemical properties of the volcanic deposit and buried soil samples are shown in Table 2. The pH of volcanic deposits was similar to that of buried soils and varied between 4.2 and 4.7, with no significant difference between the samples in 2009 and 2011. TOC and TN contents were significantly lower in the volcanic deposit samples and not significantly different among the sites, comparing with the buried soils. TOC values of buried soils varied from 48.9 g kg^−1^ for the sample from site IG3 in 2011 (sample ID, IG3-S-11) to 100.1 g kg^−1^ for the sample from site IG1 in 2011 (sample ID, IG1-S-11). In parallel with TOC, TN contents of the buried soils varied from 4.2 to 8.3 g kg^−1^. Trace amounts of total inorganic carbon (0.10 g kg^−1^) were detected for IG1-S-09, IG1-S-11, and IG2-S-09, but were undetectable in all deposit samples and the other soil samples.

*In vitro* respiration activities of the volcanic deposit samples (0.15 to 0.57 μg CO_2_-C g^−1^ h^−1^) were significantly lower than those of the buried soil samples (0.98 to 1.60 μg CO_2_-C g^−1^ h^−1^) (Tukey’s HSD test, *P* <0.05). The stable results of organic substrate utilization profiling with ECO MicroPlate were obtained by incubating the plates for 8 days (Supplementary materials, Table S1). In the case of volcanic deposits, the IG1-VD-09 sample used the fewest substrates (13 substrates) and the IG2-VD-09 sample showed the highest utilization (29 substrates). In contrast, the buried soil samples used more substrates (22–28 substrates) than the volcanic deposit samples, except for the IG1-S-09 sample (15 substrates).

### Microbial population densities

Microbial cell densities of the volcanic deposit samples ranged from about 1.0×10^8^ cell g^−1^ (dry soil) for IG1-VD-09 to 4.0×10^8^ cell g^−1^ (dry soil) for IG2-VD-09, which were about one order of magnitude lower than levels in the buried soil samples (Table 2). Similarly, plate counts on DNB were 7 to 10 times lower in the volcanic deposit samples (1.0×10^6^ to 3.0×10^6^ CFU g^−1^ [dry soil]) than those in the soil samples (7.0×10^6^ to 1.2×10^7^g^−1^ [dry soil]). This was also the case for plate counts on NB (Table 2). The plate counts on DNB were 1.2 to 1.9 times higher than those on NB for all the tested samples, except for the IG1-VD-09 sample giving 5.5 times higher counts on DNB than on NB. The difference between plate counts on DNB and NB can be explained partly by the presence of oligotrophic bacteria (48). The ergosterol content was undetectable (<0.01 μg g^−1^ [dry soil]) for all the tested volcanic deposit and buried soil samples. Because the reference soils from the forest in Miyake-jima and the arable land contained 0.09 and 0.35 μg (g dry soil)^−1^, the results indicated that the fungal populations in both the volcanic deposits and buried soils were far lower than those in normal environmental soils. For the samples taken in 2011, the fungal propagule counts were as low as 10^2^ to 10^3^ (g dry soil)^−1^ for the volcanic deposit samples and 10^3^ to 10^4^ (g dry soil)^−1^ for the buried soil samples (Table 2).

### Diversity of microbial communities

More than 200, 000 qualified reads (139,807 bacterial reads and 70,157 fungal reads), with average read lengths of 305 and 385 bp, for bacterial and fungal reads, respectively, were obtained from the Miyake-jima volcanic deposit and buried soil samples (Table S2). Community diversity, similarity, and structure were analyzed using the data rarefied at the smallest bacterial library (IG3-VD-09, 6,501 reads) and fungal library (IG1-VD-11, 4,084 reads). Total numbers of bacterial and fungal OTUs were 7,983 and 1,624, respectively. The number of bacterial OTUs per sample was in the range of 729 to 1,794, with average Good’s coverage of 90.04%, while that of fungal OTUs per sample ranged from 109 to 332, with average Good’s coverage of 97.12% (Table S2). Overall, the difference in the bacterial diversity was not related to the differences in vegetation cover and deposit age (*t*-test, *P* >0.05), but the bacterial diversity of the IG3-VD-11 sample was significantly higher than that of the other samples. As for fungal diversity, the volcanic deposit samples at site IG3 (IG3-VD-09 and IG3-VD-11) showed higher diversity than the samples form sites IG1 and IG2. The fungal diversity of the buried soil samples was clearly higher in the 2011 samples than in the 2009 samples.

### Similarities between microbial communities

**Fig. 1:**
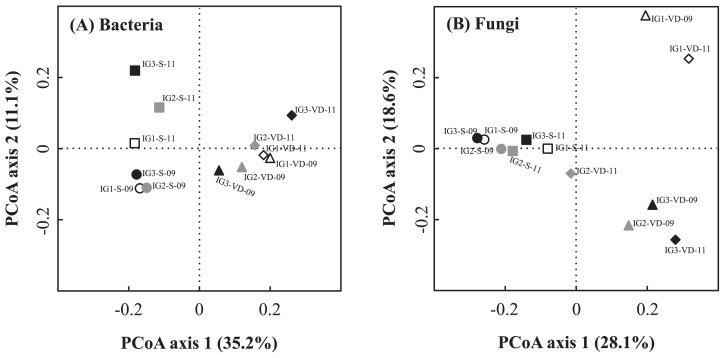
Principal coordinate analysis (PCoA) plots of bacterial (A) and fungal (B) communities of the volcanic deposit (triangles and diamonds) and soil (circles and squares) samples by weighted UniFrac. Silva bacterial and eukaryotic trees were selected as the reference trees.

PCoA plots of bacterial and fungal OTU data sets are shown in Fig. 1A and 1B, respectively. Results of this analysis showed that all bacterial communities of the volcanic deposit samples clustered away from those of the buried soil samples (Fig. 1A). The bacterial communities of the buried soil samples showed higher among-site variation than the volcanic deposit samples. As for the fungal communities, the two samples from site IG1 (IG1-VD-09 and IG1-VD-11) clustered together and this cluster was separated from the other volcanic deposit and soil samples, resulting in three clusters (Fig. 1B). The fungal communities of buried soil samples were clustered together, implying a weak influence of vegetation cover on the soil fungal community beneath the volcanic deposit.

**Fig. 2:**
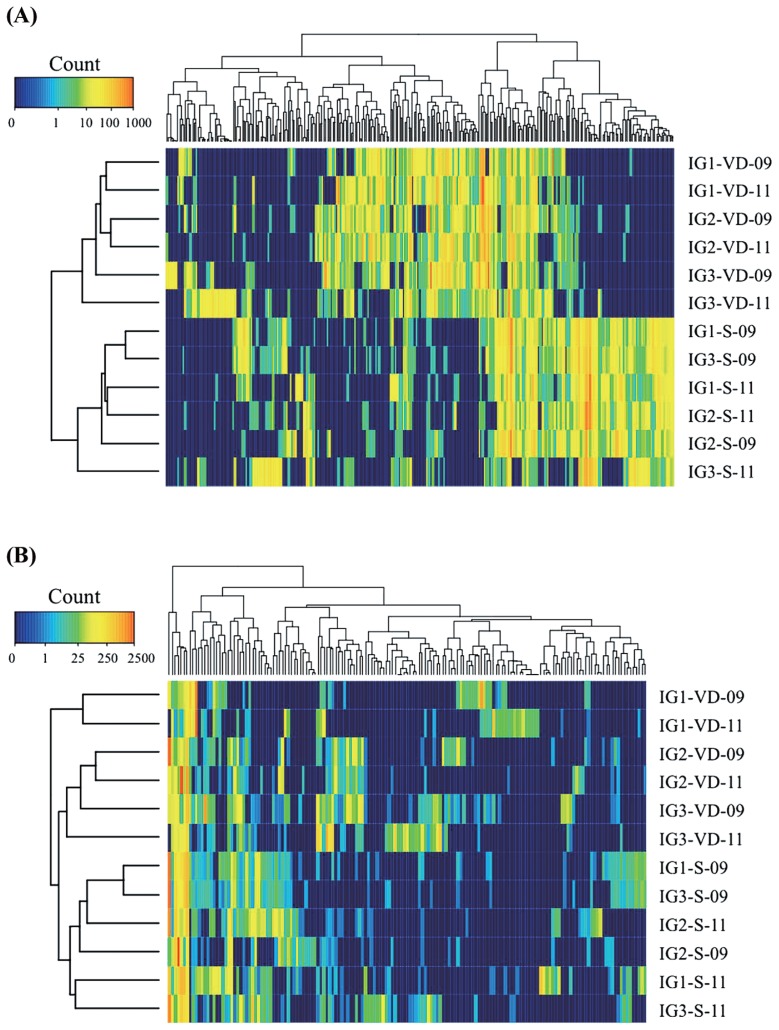
Heat map presentations of the 50 most abundant bacterial OTUs (A) and the 30 most abundant fungal OTUs (B) in each sample. The samples and OTUs were clustered on their Bray-Curtis similarities (group-average linkage). The key relates to the untransformed read counts.

The 50 most abundant bacterial OTUs in each sample were selected (275 OTUs for all 12 samples), and their abundances were compared to those in other samples, as shown in a heat map (Fig. 2A). Heat map cluster analysis showed two distinct clusters, which confirmed the difference between the bacterial communities of volcanic deposits and buried soils (Fig. 1A). This cluster analysis showed among-site variation of the bacterial community in the volcanic deposits by forming two separate clusters of site IG1 (IG1-VD-09 and IG1-VD-11) and IG2 (IG2-VD-09 and IG2-VD-11) samples and separating them from site IG3 samples (IG3-VD-09 and IG3-VD-11). On the other hand, the buried soil bacterial communities from the same sampling date but not the same site clustered together, consistent with the results of PCoA analysis (Fig. 1A).

Likewise, the 30 most abundant fungal OTUs in each sample were selected (161 OTUs for all 12 samples) and their heat map comparison is illustrated in Fig. 2B. Overall, the results of heat map cluster analysis confirmed the three major clusters given by PCoA analysis: (1) site IG1 volcanic deposit samples, (2) site IG2 and IG3 volcanic deposit samples, and (3) buried soil samples from all sites.

### Phylogenetic analysis of bacterial communities

**Fig. 3:**
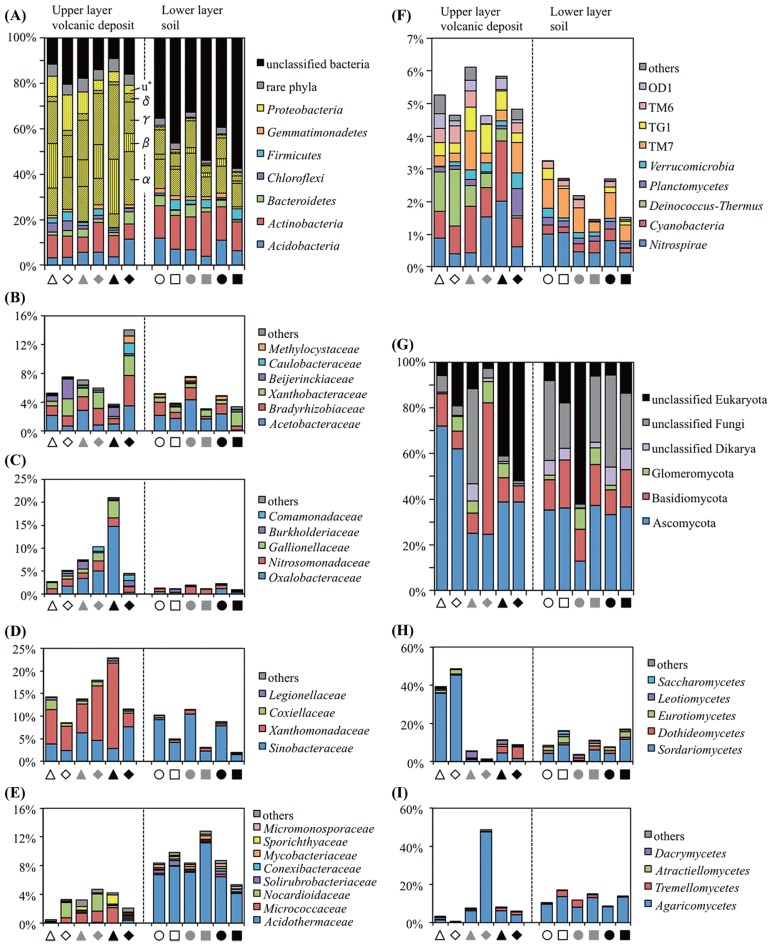
Taxonomic classification of the pyrosequencing reads. Classification at the phylum and proteobacterial class level (α, *Alphaproteobacteria; β*, *Betaproteobacteria; γ*, *Gammaproteobacteria; δ*, *Deltaproteobacteria*; u, unclassified proteobacteria) for total bacterial OTUs (A), family-level classifications of the OTUs belonging to *Alphaproteobacteria* (B), *Betaproteobacteria* (C), and *Gammaproteobacteria* (D), and *Actinobacteria* (E), and classification of low-abundance OTUs (<1% of total bacterial OTUs in each sample) into bacterial phyla (F). Classification at the phylum level for total fungal reads (G), class-level classifications of the reads of Ascomycota (H) and Basidiomycota (I). △, IG1-VD-09; 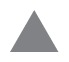, IG2-VD-09; ▲, IG3-VD-09; ⋄, IG1-VD-11; 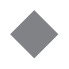, IG2-VD-11; ◆, IG3-VD-11; ○, IG1-S-09; 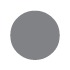, IG2-S-09; ●, IG3-S-09; □, IG1-S-11; 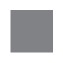, IG2-S-11; ■, IG3-S-11.

High percentages of bacterial OTUs (79.6 to 90.9%) from the volcanic deposit samples could be assigned to known bacterial phyla, while the percentages of assignable OTUs from the buried soil samples were lower (42.7 to 67.4%). Major bacterial phyla that represented >1% of each community composition were *Acidobacteria*, *Actinobacteria*, *Bacteroidetes*, *Chloroflexi*, *Gemmatimonadetes*, and *Proteobacteria* (Fig. 3A). *Proteobacteria* was the most abundant phylum in the volcanic deposit bacterial communities (50.3 to 68.4%) and constituted the major group in the buried soil communities (14.7 to 34.9%). Relative abundance estimations of the underlying classes revealed differences between the volcanic deposit and the buried soil communities. Although *Alphaproteobacteria* was predominant in the communities of both the volcanic deposit and buried soil samples (Fig. 3B), *Betaproteobacteria*, dominated by the family *Oxalobacteraceae*, and *Gammaproteobacteria*, dominated by the family *Xanthomonadaceae*, represented the main classes in the volcanic deposit communities but quite minor classes (≤1.0%) in the buried soil communities (Fig. 3C and 3D). Except for the IG3-VD-11 community, the families *Oxalobacteraceae* and *Xanthomonadaceae* increased their relative abundance in response to changes in vegetation cover from grass (site IG1) to shrub (site IG3) plants. In contrast, the IG3-VD-11 sample harbored a higher proportion of *Alphaproteobacteria* and lower proportions of *Betaproteobacteria* and *Gammaproteobacteria* than the other samples.

Further classification at the family level of the phylum *Actinobacteria* indicated that the family *Acidothermaceae* was exclusively present in all buried soil samples but not in all volcanic deposit samples (Fig. 3E). An inspection of minor bacterial populations also indicated a difference between the volcanic deposit and buried soil bacterial communities (Fig. 3F). *Deinococcus*-*Thermus* accounted for 0.4–1.7% of the total OTU number of each volcanic deposit community but <0.05% of the buried soil communities, except for IG3-VD-11. In addition, the relative abundance of *Cyanobacteria* was higher in the volcanic deposit samples (0.8–1.9%) than in the buried soil samples (0.1–0.4%).

### Phylogenetic analysis of fungal communities

Ascomycota was the most abundant phylum in the fungal communities of both volcanic deposit (24.5–72.1% of total OTUs in each sample) and buried soil (12.8–37.2%) samples, followed by Basidiomycota and Glomeromycota (Fig. 3G). It was noteworthy that the class Sordariomycetes in Ascomycota was the most abundant in site IG1 volcanic deposits (IG1-VD-09 and IG1-VD-11) but low in site IG2 and IG3 volcanic deposits (Fig. 3H). As for Basidiomycota, Agaricomycetes was the main class in the volcanic deposit, especially in the IG2-VD-11 community, and buried soil communities (Fig. 3I).

### Relationship between microbial community and environment

**Fig. 4:**
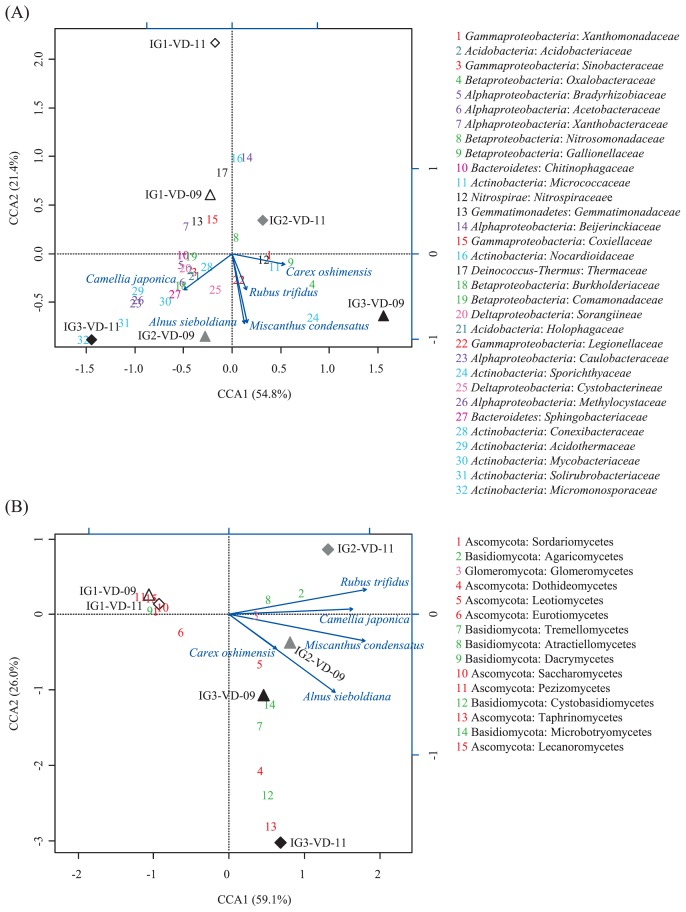
Canonical correspondence analysis (CCA) ordination plots of bacterial (A) and fungal (B) communities of six volcanic deposits (triangles and diamonds) and results of the analysis of environmental factors affecting bacterial and fungal distribution, showing significant effects of the colonizer plants. The direction of the arrows for individual plant species indicates an increasing coverage of that plant and the length of the arrows indicates the degree of correlation with the represented axes. The numbers correspond to the bacterial families (A) and fungal classes (B) in the keys on the right and are ranked according to abundance.

CCA was performed to discern possible linkages between statistically significant environmental factors, including vegetation data and known bacterial and fungal taxonomic groups. To this end, data sets of assignable OTUs to known phyla were used for analysis. For the bacterial data of volcanic deposits, the first axis separated the communities in the IG3-VD-09 and IG2-VD-11 samples from those in the others, while the second axis separated those in the IG1-VD-11, IG1-VD-09, and IG2-VD-11 from those in the others (Fig. 4A), which was in accordance with the PCoA plot data (Fig. 1A). CCA showed a positive correlation of *Oxalobacteraceae*, *Gallionellaceae*, and *Micrococcaceae* with a grass *Carex oshimensis* but a negative correlation of *Xanthobacteraceae* and *Gemmatimonadaceae* with the grass. The presence of *Sphingobacteriaceae*, *Burkholderiaceae*, and *Acetobacteraceae* correlated positively with a tree, *Camellia japonica*. No strong positive correlation was found between any bacterial families and the most abundant grass, *Miscanthus condensatus* and the most abundant shrub, *Alnus sieboldiana*, but *Thermaceae* and *Coxiellaceae* showed a negative correlation with these plants. No strong influence of the chemical properties (pH, TOC, and TN) on bacterial community was found, as expected from the low among-site variation of the chemical properties (Table 2).

For the fungal data of volcanic deposits (Fig. 4B), the first axis separated the communities in site IG1 samples (IG1-VD-09 and IG1-VD-11) from those in site IG2 and IG3 samples, and the second axis separated site IG1 samples and IG2-VD-11 samples from those in the others, all consistent with the PCoA results (Fig. 1B). Fungi that thrived in the site IG1 volcanic deposit, such as Sordariomycetes, Saccharomycetes, Pezizomycetes, and Lecanoromycetes in the phylum Ascomycota, and Dacrymycetes in the phylum Basidiomycota showed a highly negative relationship with the major plants, *Miscanthus condensatus* and *Alnus sieboldiana*. Agaricomycetes in the phylum Basidiomycota correlated positively but Eurotiomycetes in the phylum Ascomycota negatively with a shrub, *Rubus trifidus*.

In the case of buried soils, *Nocardioidaceae* showed a positive correlation with the major grass, *Miscanthus condensatus* but *Oxalobacteraceae* and *Coxiellaceae* showed a negative correlation with the major grass (Fig. S4). *Beijerinckiaceae*, *Acetobacteraceae*, and *Micrococcaceae* correlated negatively with *Carex oshimensis* and *Alnus sieboldiana*. In the fungal data, only negative correlation of Tremellomycetes with *Alnus sieboldiana* and *Carex oshimensis* was found (Fig. S4B). TOC and TN seemed to have no important impact on known bacterial and fungal groups (Fig. S4A and S4B).

## Discussion

A number of studies have indicated that the early development of the microbial community on recent volcanic deposits (11, 42, 44, 60) and deglaciated soils (45, 56, 57) was associated with pioneer colonizer plants. In a study of a volcanic desert on Mount Fuji, total carbon (TC), TN, and soil organic matter (SOM) contents increased with vegetation development, and soil microbial biomass was strongly correlated with TC, TN, and SOM contents (60). These findings suggested that the belowground accumulation of organic nutrients along with vegetation development was a determinant of soil microbial biomass. At our study sites, sites IG2 and IG3 were covered fully with grass plants and partly or mostly with shrub plants. In spite of vegetation development, the TOC values of all volcanic deposit samples (0.2–0.7 g kg^−1^ in Table 2) were much lower than those of the samples in the above-mentioned studies [8.1–28.9 g kg^−1^ for a subalpine volcanic desert on Mount Fuji (60); 2.5–3.0 g kg^−1^ for a glacier forefield (33)]. From the data of the 16-, 38-, 60-, and 125-year-old Volcanogenous Regosols on Miyake-jima (27), a positive linear relationship was noted between volcanic deposit age and TC, which is approximated by an equation, TC (g kg^−1^) = 0.023 *t* (*r*^2^ = 0.98), where *t* is volcanic deposit age (y). The TOC values (0.2–0.7 g kg^−1^) of our volcanic deposit samples (age, 9–11 y; total inorganic carbon, undetectable levels) substantially fit the equation. From the equation, it will take >43 y to accumulate TC >1.0 g kg^−1^ in the volcanic deposit. Therefore, our study reveals the earliest change in the belowground microbial community at the onset of vegetation cover development.

**Fig. 5:**
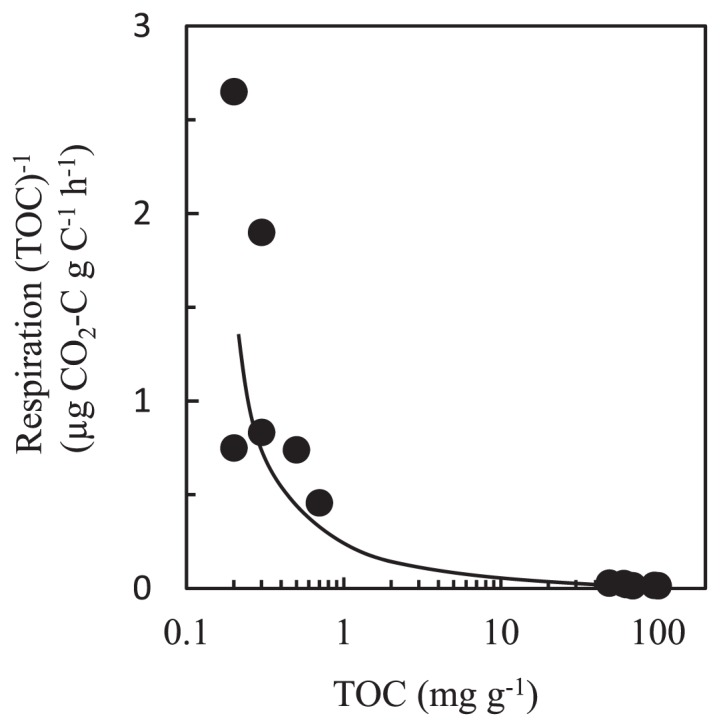
Relationship between TOC and respiration per unit amount of organic carbon (TOC).

Although the volcanic deposit samples contained approx. 100 times lower TOC than the buried soil samples, differences in microbial population density were not as large as the differences in TOC values (Table 2). This can be explained partly by differences in the content of available organic matter. When the *in vitro* respiratory activity of all samples is plotted against the corresponding TOC values, the respiration per unit of organic carbon decreased sharply at higher TOC values (Fig. 5), suggesting a relative reduction in available substrate in the samples. This organic matter dynamics was noted previously by a study of 18- to 300-year-old Hawaiian volcanic deposits (29). Interestingly, a recent study of deglaciated soils showed that soil carbon along the chronosequence was of microbial origin and inputs of organic matter were dominated by microbial carbon and nitrogen fixation (56). Generally, microbial biomass is characterized by a low C:N ratio and biomass debris is readily consumable for soil microbes, which can result in high activity of respiration per unit of organic carbon. Indeed, the C:N ratio was much lower in the volcanic deposit samples (0.4 to 1.6) than the buried soil samples (11.8 to 13.6) (Table 2).

Various preceding studies on volcanic deposits, deglaciated soils, and other newly exposed minerals have shown that the phylum *Proteobacteria* usually dominates the early bacterial community (33, 42, 64), because the bacteria in this phylum have advantageous traits such as phototrophy, photoheterotrophy, and chemolithotrophy, in early ecosystems with limited nutrient resources. Our results showed that although the vegetation developed at different levels, *Proteobacteria* was still the most abundant phylum in the bacterial community of volcanic deposits. Inspection of the underlying families revealed the predominance of the family *Oxalobacteraceae* in *Betaproteobacteria* and *Xanthomonadaceae* in *Gammaproteobacteria* in the volcanic deposit communities (Fig. 3C and 3D). The family *Oxalobacteraceae* was reported as root-colonizing heterotrophic bacteria in a succession of bacterial communities during early plant development (13, 14). This can be expected to be true for the bacterial community of the Miyake-jima volcanic deposit because CCA showed a positive correlation of *Oxalobacteraceae* with a grass *Carex oshimensis* (Fig. 4A). The family *Xanthomonadaceae* was reported as a major component of pasture rather than woodland or broad-leaved forest (6). In our data, the correlation of *Xanthomonadaceae* with *Carex oshimensis* also seemed to be positive in the CCA plot (Fig. 4A). In the IG3-VD-11 samples, *Oxalobacteraceae* and *Xanthomonadaceae* were replaced by *Alphaproteobacteria*, specifically the families *Acetobacteraceae*, *Bradyrhizobiaceae*, and *Xanthobacteraceae* (Fig. 3B to 3D). This succession seems to be supported by the notion that plants raised the proportion of *Alphaproteobacteria*, particularly *Rhizobiales*, in various soil environments (16, 33).

*Actinobacteria* are the second most abundant phylum dominating the bacterial community in volcanic deposits (Fig. 3A and 3E), which are generally known to decompose recalcitrant polymers in soils (17). A recent study of Zimmerman sand created by glacial outwash indicated that host plant species and increasing plant richness altered the composition of *Streptomyces* communities, which were the most abundant among the *Actinobacteria* (1). Similar to this finding, in our study, the family-level composition of *Actinobacteria* in the volcanic deposits was also found to differ distinctly at different sites (Fig. 3E). On the other hand, *Actinobacteria* composition in the buried soil samples was essentially invariant among the sites. As discussed above, the respiration per unit of soil organic carbon seems to reflect the nature of soil organic matter and thus the very low respiration rates of the Miyake-jima buried soil samples suggest the presence of higher amounts of recalcitrant substrates than those of readily consumable substrates in the soil samples. This may explain the steady population density of *Actinobacteria* in the buried soil.

The phylum *Acidobacteria* is ubiquitous and abundant in various soil environments (22, 37), but the proportion of this group is relatively low in early bacterial communities (11, 33, 42, 64). In the case of Miyake-jima volcanic deposits, the proportion of *Acidobacteria* increased from 3.5% to 11.7% with vegetation development (Fig. 3A), but was still lower than the average level (20%) in various soils (21). It has been reported that the abundance and composition of *Acidobacteria* in soils seemed to be strongly regulated by the soil pH value (22). As mentioned above, the volcanic deposit samples displayed low among-sample variation for the pH (4.2 to 4.7), and thus the environmental pH is not likely to be the determinative factor for the distribution of *Acidobacteria* in Miyake-jima volcanic deposits.

Our results showed that the phylum *Cyanobacteria* was present at low levels (0.8–1.9% of bacterial communities) in the Miyake-jima volcanic deposits (Fig. 3F). This organism is known to be the primary colonizer of newly exposed minerals and dominates early microbial communities (11, 19). In addition to this phototroph, the filamentous anoxygenic phototrophic *Chloroflexi* was found to be present at higher levels (about 4%) in the site IG1 volcanic deposit than *Cyanobacteria* (Fig. 3A). Although the population was low in site IG2 and IG3 deposits, the *Chloroflexi* group may occupy a wider niche than *Cyanobacteria* in unvegetated sites. It is also noted that *Deinococcus-Thermus* was found in IG1 volcanic deposits but not in soil samples (Fig. 3F). The genus *Deinococcus* is known to show marked resistance to a range of damage caused by ionizing radiation, desiccation, UV radiation, and oxidizing agents (41). Resistance to solar radiation or desiccation may be a secondary determinative factor for the survival of microbes in volcanic deposits, particularly at unvegetated sites.

No detectable amount of ergosterol and low counts of fungal propagules in the Miyake-jima volcanic deposit and buried soil samples (Table 2) indicated that fungi constituted low fractions of their microbial communities. From the contents of ergosterol in upland soils (about 1.0 to 2.2 μg ergosterol [g dry soil]^−1^) (62), the fungal population density of the Miyake-jima samples is estimated to be less than one-tenth of those in the upland soils. It has been reported that fungi are more influenced by vegetation type than prokaryotes, because fungi are directly associated with plants (46). In spite of the low fungal populations, a substantial number of fungal reads were obtained from the volcanic deposit samples. The results of PCoA showed high among-site variation for the fungal communities in the volcanic deposits (Fig. 1B). Further, the CCA plot showed Ascomycota thriving in the site IG1 volcanic deposit; for example, the class Sordariomycetes showed a highly negative relationship with the major plants, *Miscanthus condensatus* and *Alnus sieboldiana* (Fig. 4B). Interestingly, Zumsteg *et al.* reported a similar observation, the succession from an Ascomycota-dominated community in unvegetated soils to a more Basidiomycota-dominated community in vegetated soils in the forefield of the Damma glacier (64). Members of Sordariomycetes are ubiquitous and represent pathogens and endophytes of plants, animal pathogens, and mycoparasites (61). However, information on the Sordariomycetes in unvegetated volcanic deposits is not yet available. Within Basidiomycota, the class Agaricomycetes was exclusively present in the IG2-VD-11 sample (Fig. 3I). Members of Agaricomycetes are the most important ectomycorrhizal fungi, which construct symbiotic associations with many territorial plants (18). Further, investigations at the glacier forefield showed that plant colonization could increase the proportion of mycorrhizal fungi in the early soil communities of alpine habitats (40, 64). Analogously, in our results, Agaricomycetes showed a positive correlation with a shrub, *Rubus trifidus* (Fig. 4B). To examine further these fungus-plant correlations, community analyses should focus on the rhizosphere of colonizer plants.

In conclusion, this study showed that microbial communities in recent Miyake-jima volcanic deposits were phylogenetically diverse, despite low-carbon conditions. Because the volcanic deposit samples displayed low among-site variation for chemical properties (pH, TOC, and TN), there is no apparent factor other than the aboveground vegetation cover to explain the difference in microbial community among the different site volcanic deposits. Indeed, CCA could show several positive and negative relationships between microbial groups and plant species. Our findings give a better understanding of how belowground microbial communities develop and interact with the establishment of the first aboveground plants in newly exposed volcanic deposits.

## Supplemental material



## Figures and Tables

**Table 1:** Vegetation cover profiles at sites IG1, IG2, and IG3 on Miyake-jima*

Site	Date	Low grass layer		High grass layer		Shrub layer		Total No. of plant species	Major plants other than *Miscanthus condensatus*
		
		Height (m)	Coverage (%)	Height (m)	Coverage (%)	Height (m)	Coverage (%)		
IG1	2009	1	20	—	—	—	—	5	
	2011	1	45	—	—	—	—	6	*Polygonum cuspidatum* var. *terminale* (grass)
IG2	2009	0.5	10	3	100	6	15	15	*Alnus sieboldiana* (shrub)
	2011	0.5	20	3	100	6	25	14	*Rubus trifidus* (shrub), *Alnus sieboldiana* (shrub)
IG3	2009	0.7	20	3	100	6.5	65	21	*Carex oshimensis* (grass), *Alnus sieboldiana* (shrub)
	2011	0.5	30	3	90	6.5	60	21	*Alnus sieboldiana* (shrub)

* The height and percentage cover of each layer were recorded. *Miscanthus condensatus* (grass) was dominant at all sites. —, not detected.

**Table 2:** Chemical and microbiological properties of Miyake-jima volcanic deposits and soils*

Sample ID†	pH	TOC (g kg^−1^)	TN (g kg^−1^)	C:N ratio	Water content (%)	*In vitro* respiratory activity (μg CO_2_-C g^−1^ h^−1^)	TDC (×10^9^ cells g^−1^)	Bacterial plate count (×10^6^ CFU g^−1^) on		Fungal propagule (×10^3^ g^−1^)
								DNB	NB	
Volcanic deposit										
IG1-VD-09	4.3	0.2 ± 0.0^a^	0.2 ± 0.0^a^	0.9	22	0.15 ± 0.02^a^	0.10 ± 0.01^a^	1.01 ± 0.17^a^	0.13 ± 0.01^a^	ND
IG1-VD-11	4.7	0.2 ± 0.0^a^	0.1 ± 0.0^a^	1.6	25	0.53 ± 0.07^abc^	0.39 ± 0.02^a^	1.26 ± 0.06^a^	0.68 ± 0.08^a^	0.60 ± 0.09^a^
IG2-VD-09	4.3	0.5 ± 0.1^a^	0.9 ± 0.0^a^	0.6	21	0.37 ± 0.11^abc^	0.45 ± 0.05^a^	3.37 ± 0.56^ab^	1.73 ± 0.18^a^	ND
IG2-VD-11	4.5	0.3 ± 0.1^a^	0.3 ± 0.1^a^	0.8	26	0.57 ± 0.10^abc^	0.42 ± 0.01^a^	1.18 ± 0.05^a^	0.59 ± 0.04^a^	0.44 ± 0.03^a^
IG3-VD-09	4.2	0.3 ± 0.0^a^	0.8 ± 0.1^a^	0.4	23	0.25 ± 0.05^ab^	0.20 ± 0.02^a^	1.22 ± 0.07^a^	0.86 ± 0.07^a^	ND
IG3-VD-11	4.4	0.7 ± 0.0^a^	0.5 ± 0.1^a^	1.3	27	0.32 ± 0.06^abc^	0.29 ± 0.03^a^	1.03 ± 0.05^a^	0.85 ± 0.11^a^	2.84 ± 0.18^ab^
Buried soil										
IG1-S-09	4.5	94.5 ± 4.3^d^	7.3 ± 0.1^d^	12.9	50	0.98 ± 0.16^bcde^	1.60±0.15^b^	11.2 ± 0.9^de^	6.90 ± 0.52^cd^	ND
IG1-S-11	4.3	100.1 ± 1.5^d^	8.3 ± 0.6^d^	12.1	55	1.56 ± 0.19^e^	2.23±0.11^c^	11.7 ± 1.1^e^	8.14 ± 0.92^d^	16.82 ± 0.54^d^
IG2-S-09	4.4	69.0 ± 7.5^c^	5.3 ± 0.2^c^	12.9	45	1.06 ± 0.03^cde^	1.75±0.13^b^	9.1 ± 1.1^cd^	7.50 ± 0.79^cd^	ND
IG2-S-11	4.5	60.3 ± 7.1^bc^	5.1 ± 0.3^bc^	11.9	46	1.60 ± 0.28^e^	1.85±0.11^bc^	7.6 ± 0.8^cd^	5.46 ± 0.51^bc^	9.06 ± 1.90^c^
IG3-S-09	4.4	62.6 ± 2.2^bc^	4.6 ± 0.2^bc^	13.6	52	1.37 ± 0.17^de^	1.93±0.13^bc^	9.3 ± 1.1^cde^	7.95 ± 0.70^d^	ND
IG3-S-11	4.6	48.9 ± 0.8^b^	4.2 ± 0.1^b^	11.8	44	1.41 ± 0.30^e^	1.95±0.08^bc^	7.0 ± 0.8^bc^	4.48 ± 0.32^b^	4.87 ± 0.61^b^

* TOC, total organic carbon; TN, total nitrogen; TDC, total direct count; DNB, 1:100 diluted nutrient broth; NB, nutrient broth; ND, not determined. C:N ratio, the ratio of the sum of TOC and inorganic carbon to TN. Data for TOC, TN, respiratory activity and TDC represent the mean and standard deviation (STD) of triplicate determinations and those for the plate counts the mean and STD of four replicate plates. Different letters indicate significant differences between mean values within a given comparison (MANOVA with Tukey’s HSD test, *P* <0.05).

† −09, sampled in 2009; −11, sampled in 2011.
