# Supplementary material for: Characterization of Early Microbial Communities on Volcanic Deposits along a Vegetation Gradient on the Island of Miyake, Japan
Source: Microbes Environ. 2014 Jan 24;29(1):38–49. doi: 10.1264/jsme2.ME13142 (PMC4041228; doi:10.1264/jsme2.ME13142)
Supplement: Supplementary file 1 [file 29_38_1.pdf]

**Supplemental material**

Detailed methods

*1. Metrological and geological information about Miyake-jima and the eruption in 2000*
Miyake-jima is characterized by a humid warm-temperate climate. The meteorological information is also available at the MetBroker Weather Station 476770 (http://www.tutiempo.net/en/Climate/MIYAKEJIMA/476770.htm). The island is exposed to strong winds (annual wind speed, 5.1 m/s) with frequent wind directions in southwest, west-southwest, and northeast. The eruption occurred at Mt. Oyama, the summit of the island, from July to September, 2000, ejecting large amounts of volcanic ash and finally forming a large collapsed crater (1.6 km diameter; 500 m deep) (1). An extremely large amount of volcanic gas including $SO_2$ has been released since mid-August 2000 after the formation of the summit crater. According to the report by Kazahaya *et al.* (2), in December 2000, the $SO_2$ emission rate averaged for the month peaked at 54 kt $d^{-1}$ and the emission rate gradually decreased, almost linearly when plotted on a log scale, to 7 kt $d^{-1}$ by the end of 2002. The total volume of tephra deposits in the 2000 eruptive event was about $9.3\times10^6$ $m^3$ dense-rock equivalent to about $2.3\times10^{10}$ kg (3). An on-site vegetation survey in 2003 showed that vegetation recovery was found in the windward area and characterized by stem sprout of defoliated trees, sprout from buried vegetative organ, germination of buried seeds, and colonization of seeds and spores (4).

*2. PCR primer sequences*
The V1-V2 region in the 16S ribosomal RNA gene was amplified using universal primers 27Fmod (5'-ANNNNNNNNNNagrgtttgatymtggctcag-3') and 338R (5'-Btgctgcctcccgtaggagt-3'), where A and B represent the adaptors A and B, respectively, for 454 pyrosequencing (5). The NNNNNNNNNN indicates a unique 10-bp barcode sequence for each sample. The 27Fmod primer originated from 27F by changing the third base A in 27F sequence to R. Using 27Fmod in preparation of 16S rDNA amplicons gave more accurate results for evaluation of the bacteria composition than using 27F by improving underestimation of the genus *Bifidobacterium* often observed on using primer 27F (6). PCR was performed in 1×Ex Taq PCR buffer (50 µL), deoxynucleoside triphosphate (2.5 mM), Ex Taq polymerase (Takara Bio, Otsu, Japan), each primer (10 µM), and 40 ng of extracted DNA. Another universal primers set 817F (5'-ANNNNNNNNNNttagcatggaataatrraatagga-3') and 1196R (5'-Btctggacctggtgagtttcc-3') were performed for analyzing the fungal community (7), where same pyrosequencing adaptors and barcode sequences to amplification of 16S rRNA gene were used.

References


1. Nakada, S., M. Nagai, M. Yasuda, T. Shimano, N. Geshi, M. Ohno, T. Akimasa, T. Kameko, and T. Fujii. 2001. Chronology of the Miyakejima 2000 eruption: characteristics of summit collapsed crater and eruption products. J. Geogr. (Chigaku Zassi) 110:168-180 (in Japanese).
2. Kazahaya, K., H. Shinohara, K. Uto, M. Odai, Y. Nakahori, H. Mori, H. Iino, M. Miyashita, and J. Hirabayashi. 2004. Gigantic $SO_2$ emission from Miyakejima volcano, Japan, caused by caldera collapse. Geology 32:425-428.

3. Nakada, S., M. Nagai, T. Kaneko, A. Nozawa, and K. Suzuki-Kamata. 2005. Chronology and products of the 2000 eruption of Miyakejima Volcano, Japan. Bull. Volcanol. 67:205-218.
4. Kamijo, T., and K. Hashiba. 2003. Island ecosystem and vegetation dynamics before and after the 2000-year eruption on Miyake-jima Island, Japan, with implications for conservation of the island's ecosystem. Global Environ. Res. 7:69-78.
5. Kim, S.-W., W. Suda, S. Kim, K. Oshima, S. Fukuda, H. Ohno, H. Morita, and M. Hattori. 2013. Robustness of gut microbiota of healthy adults in response to probiotic Intervention revealed by high-throughput pyrosequencing. DNA Res. 20:241-253.
6. Hattori, M., and T. D. Taylor. 2009. The human intestinal microbiome: a new frontier of human biology. DNA Res. 16:1-12.
7. Borneman, J., and R. J. Hartin. 2000. PCR primers that amplify fungal rRNA genes from environmental samples. Appl. Environ. Microbiol. 66:4356-4360.

**Table S1.** ECO MicroPlate reactions for water extracts from the Miyake-jima volcanic deposits and soils

| Substrate | | Volcanic deposit at site[a]: | | | Soil at site[a]: | | |
|---|---|---|---|---|---|---|---|
| | | IG1 | IG2 | IG3 | IG1 | IG2 | IG3 |
| Amines | Phenyl ethylamine | -/+ | +/+ | w/w | -/+ | +/+ | +/+ |
| | Putrescine | -/- | +/w | w/- | -/+ | +/+ | w/+ |
| Amino acids | L-Arginine | w/+ | +/+ | w/+ | w/+ | +/+ | +/+ |
| | L-Asparagine | -/+ | +/+ | w/+ | -/+ | +/+ | +/+ |
| | Glycyl-L-Glutamic acid | -/- | +/+ | w/- | -/+ | w | +/+ |
| | L-Phenylalanine | +/+ | +/+ | +/+ | -/+ | +/+ | +/+ |
| | L-Serine | -/+ | +/+ | w/w | w/+ | +/w | +/+ |
| | L-Threonine | w/w | +/+ | -/+ | -/+ | +/+ | +/+ |
| Carbohydrates | D-Cellobiose | -/w | +/+ | -/- | w/+ | +/+ | +/+ |
| | Erythritol | -/- | w/+ | -/- | -/+ | -/+ | w/- |
| | D-Galactonic acid, γ-lactone | +/+ | +/+ | w/+ | w/+ | +/+ | w/+ |
| | N-Acetyl-D-Glucosamine | w/w | +/+ | w/+ | w/+ | +/w | +/+ |
| | Glucose-1-Phosphate | -/- | w/w | -/- | -/- | -/- | -/+ |
| | β-Methyl-D-Glucoside | -/w | +/+ | -/w | -/- | -/w | w/w |
| | D,L-α-Glycerol phosphate | w/- | +/- | -/w | -/+ | w/- | -/+ |
| | α-D-Lactose | -/w | w/+ | w/- | w/+ | +/w | +/w |
| | D-Mannitol | -/+ | +/+ | +/w | w/+ | +/w | +/+ |
| | D-Xylose | -/- | -/- | -/- | w/- | w/- | -/- |
| Carboxylic acids | D-Galacturonic acid | w/+ | +/+ | w/+ | -/+ | +/- | +/+ |
| | D-Glucosaminic acid | -/w | +/+ | +/- | -/+ | +/w | +/+ |
| | γ-Hydroxybutyric acid | w/+ | +/+ | w/w | -/+ | w/- | -/w |
| | α-Ketobutyric acid | -/+ | +/+ | w/w | -/- | +/- | +/+ |
| | Itaconic acid | -/+ | w/+ | -/- | -/+ | -/- | -/w |
| | D-malic acid | +/- | +/+ | w/+ | -/+ | +/w | +/+ |
| | Pyruvic acid methyl ester | +/+ | +/+ | +/+ | +/+ | +/+ | +/+ |
| Phenolic compounds | 2-Hydroxy benzoic acid | w/- | +/+ | w/- | w/+ | -/- | -/w |
| | 4-Hydroxy benzoic acid | w/+ | +/w | w/- | +/+ | +/- | +/+ |
| Polymers | α-Cyclodextrin | -/- | w/- | -/- | w/- | -/+ | w/- |
| | Glycogen | -/- | -/- | -/- | w/- | -/+ | -/+ |
| | Tween 40 | -/+ | +/+ | -/+ | +/+ | +/+ | +/+ |
| | Tween 80 | w/+ | +/+ | +/+ | +/+ | +/+ | +/+ |
| Positive reaction | | 4/15 | 14/24 | 5/11 | 4/25 | 20/14 | 19/23 |
| Weakly positive reaction | | 9/6 | 5/3 | 15/7 | 11/0 | 4/8 | 5/5 |
| Total No. of utilized substrates | | 13/21 | 29/27 | 20/18 | 15/25 | 24/22 | 24/28 |

[a] Reactions were scored positive (+), weakly positive (w), or negative (−) relative to control wells. Assays were examined separately in 2009 and 2011 (2009 sample/2011 sample).

**Table S2.** Qualified reads, OTU counts, alpha diversity indexes of microbial communities, and Good’s coverage*

| Sample ID | Bacteria | | | | | | Fungi | | | | | |
|---|---|---|---|---|---|---|---|---|---|---|---|---|
| | No. of qualified reads | OTU count | ACE | *H′* | 1/*D* | C (%) | No. of qualified reads | OTU count | ACE | *H′* | 1/*D* | C (%) |
| IG1-VD-09 | 35,835 | 846 | 2,218 | 5.04 | 39.49 | 93.26 | 4,616 | 166 | 365 | 2.44 | 4.79 | 98.14 |
| IG1-VD-11 | 8,310 | 757 | 1,731 | 4.88 | 39.25 | 94.15 | 4,084 | 187 | 640 | 2.44 | 3.80 | 97.58 |
| IG2-VD-09 | 9,073 | 1,207 | 4,346 | 5.68 | 99.52 | 89.16 | 5,283 | 210 | 698 | 2.61 | 4.21 | 97.14 |
| IG2-VD-11 | 9,732 | 729 | 1,778 | 4.93 | 53.89 | 94.34 | 5,912 | 134 | 471 | 1.96 | 3.12 | 98.11 |
| IG3-VD-09 | 6,501 | 824 | 2,493 | 4.59 | 22.05 | 92.97 | 7,511 | 291 | 913 | 3.71 | 15.17 | 96.23 |
| IG3-VD-11 | 12,256 | 1,794 | 5,579 | 6.50 | 208.51 | 83.99 | 8,741 | 332 | 1,016 | 3.73 | 16.26 | 95.64 |
| IG1-S-09 | 7,396 | 1,086 | 3,075 | 5.54 | 67.93 | 90.88 | 4,779 | 212 | 617 | 2.67 | 5.12 | 97.21 |
| IG1-S-11 | 9,336 | 1,276 | 4,257 | 5.62 | 68.88 | 88.36 | 7,992 | 242 | 1,172 | 3.38 | 14.02 | 96.55 |
| IG2-S-09 | 9,348 | 1,116 | 3,631 | 5.45 | 57.83 | 90.14 | 5,250 | 109 | 218 | 1.79 | 2.66 | 98.53 |
| IG2-S-11 | 9,844 | 1,254 | 4,621 | 5.60 | 63.69 | 88.48 | 6,080 | 244 | 909 | 3.14 | 8.45 | 96.45 |
| IG3-S-09 | 9,265 | 1,166 | 3,665 | 5.59 | 72.53 | 89.88 | 4,971 | 171 | 426 | 2.29 | 3.93 | 97.85 |
| IG3-S-11 | 12,911 | 1,549 | 6,243 | 5.86 | 93.91 | 84.88 | 4,938 | 280 | 1,059 | 3.18 | 8.04 | 95.98 |

*Samples were rarefied at the smallest bacterial library (IG3-VD-09, 6501 reads) and fungal library (IG1-VD-11, 4084 reads) for OTU count and statistical analyses. All indexes were calculated at cutoff level of 0.03 in the average neighbor method. ACE, abundance-based coverage estimator; *H′*, Shannon index; *1/D*, inverse Simpson index; C, Good’s coverage.

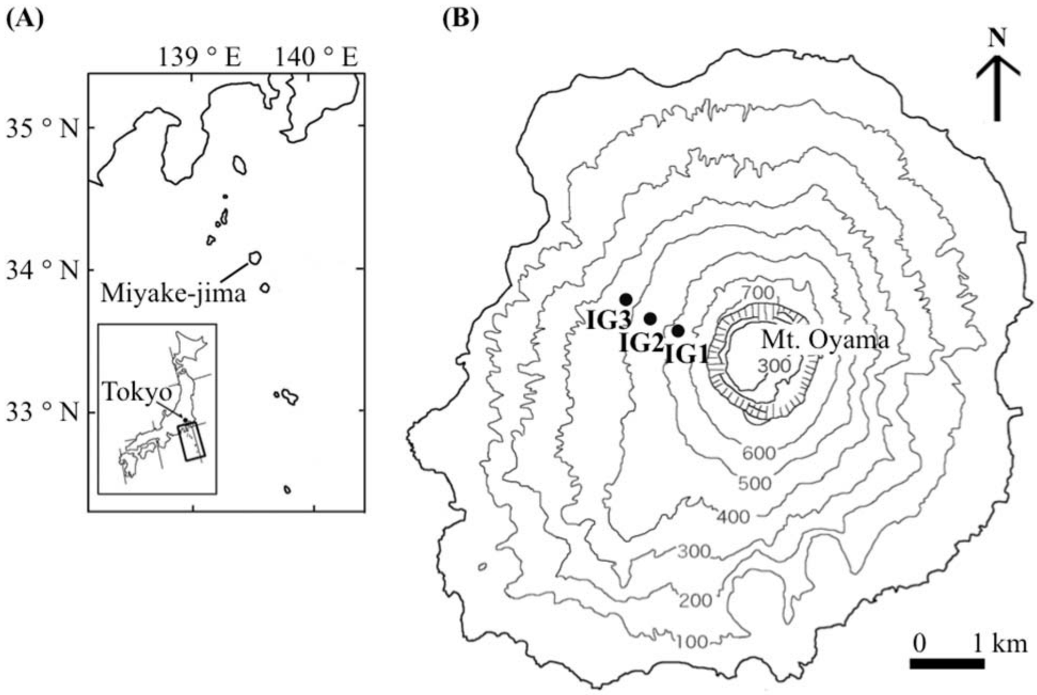


**Fig. S1.** Maps showing the location of the Island of Miyake in the western rim of the Pacific Ocean (A) and the sampling sites (B).

(A)

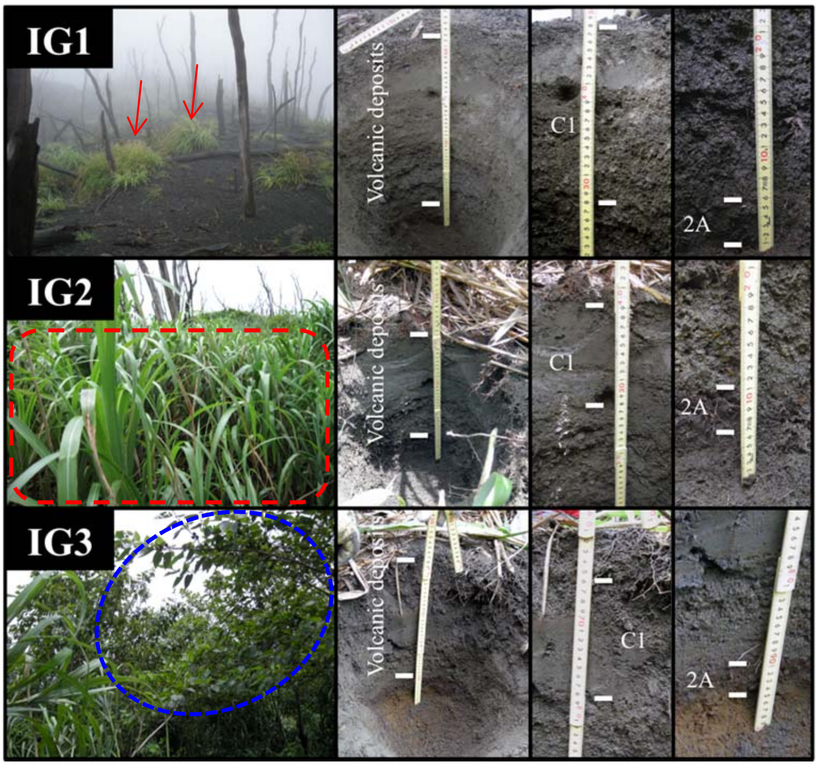


(B)

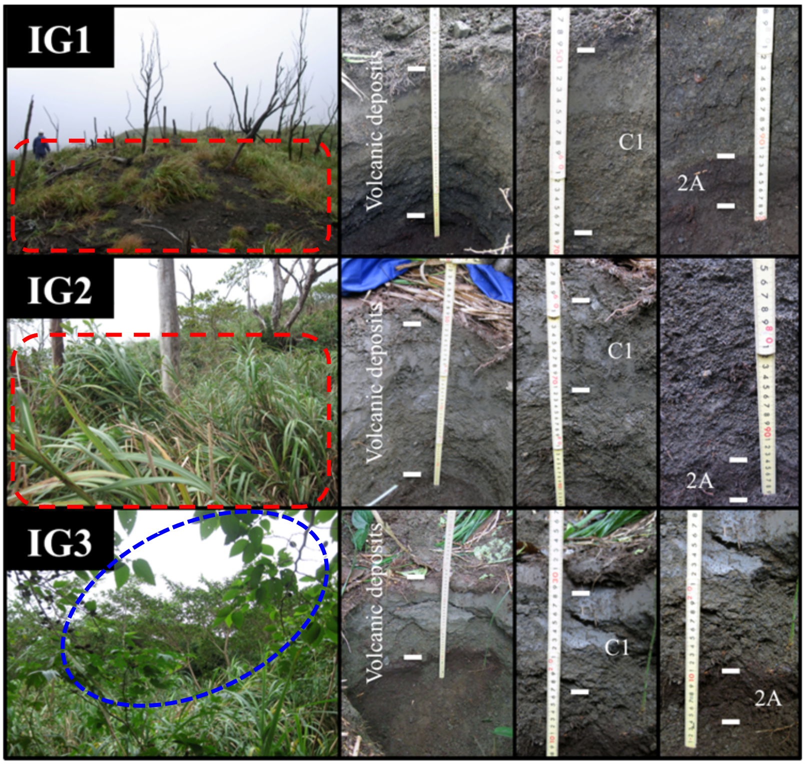


**Fig. S2.** Photographs showing the vegetation cover and soil layer profiles of volcanic deposit (C1) and buried soil (2A) at sites IG1, IG2, and IG3 in 2009 (A) and 2011 (B).
The leftmost photographs show the dominant plants, *Miscanthus condensatus* [red arrows in IG1 (panel A) and red broken squares in IG1 (panel B) and IG2 (panels A and B)] and *Alnus sieboldiana* [blue broken circles in IG3 (panels A and B)].

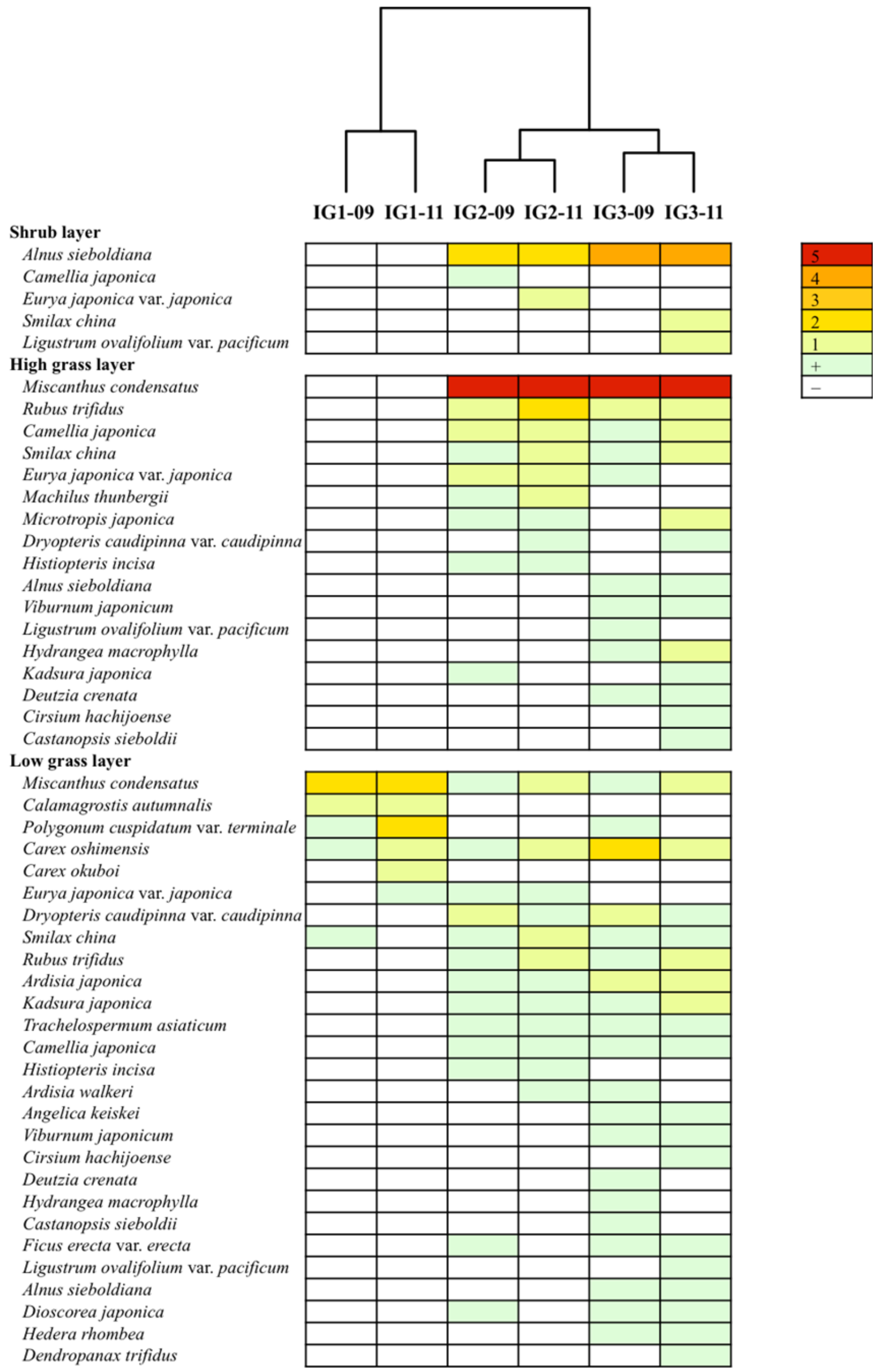


**Fig. S3.** Heat map showing the abundances and distributions of plant species at sites IG1, IG2, and IG3.

The vegetation was stratified into shrub, high grass, and low grass layers. The color key relates to species abundance represented in Braun-Blanquet cover-abundance scale: 5, c ≥ 75%; 4, 50% ≤ c ≤ 75%; 3, 25% ≤ c ≤ 50%; 2, 5% ≤ c ≤ 25%; 1, 0.1% ≤ c ≤ 5%; +, c ≤ 0.1%; -, not detected.

(A)

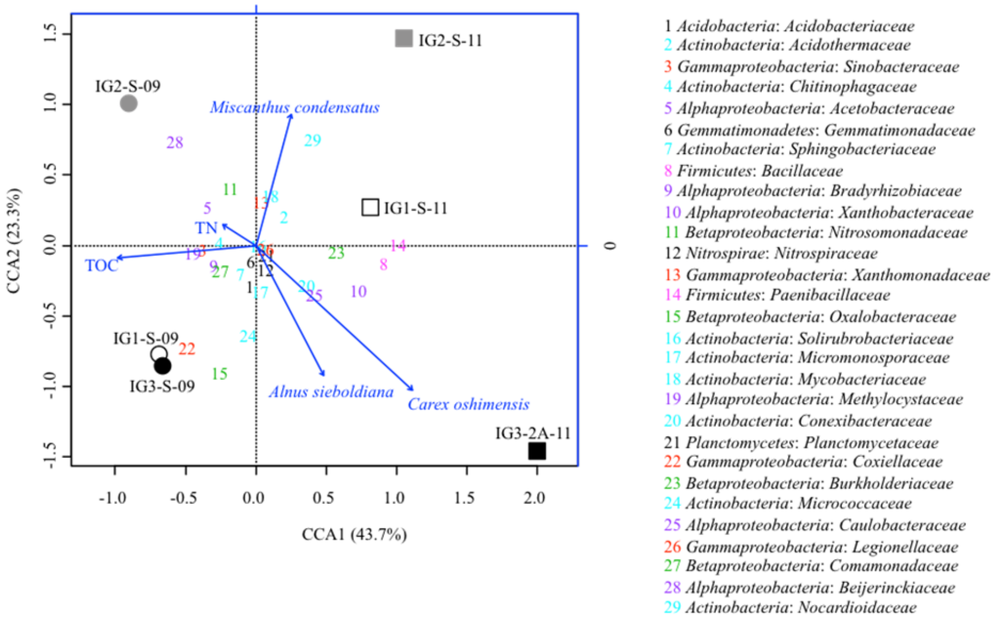


(B)

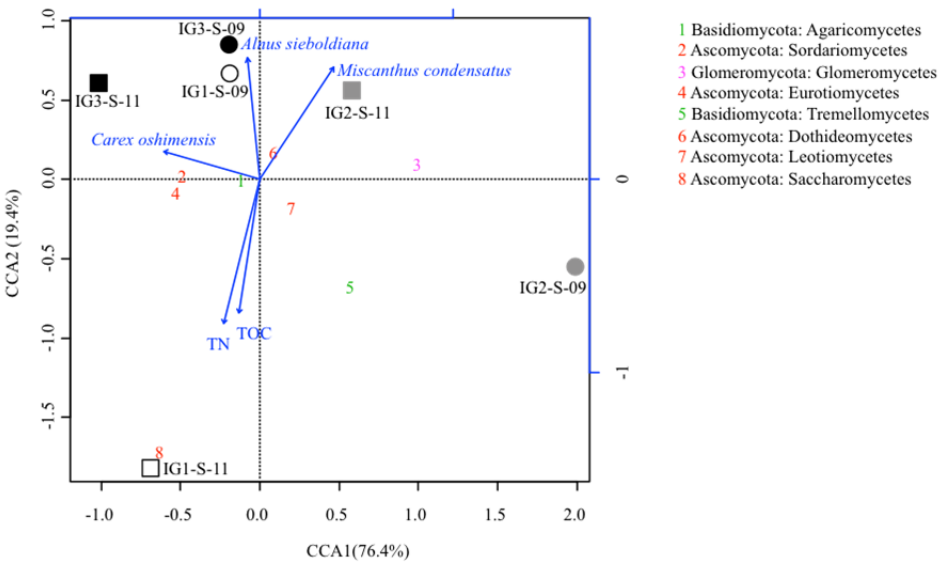


**Fig. S4.** Canonical correspondence analysis (CCA) ordination plots of bacterial (A) and fungal (B) communities of six buried soil samples (circles and squares) and results of the analysis of environmental factors affecting bacterial and fungal distribution, showing effects of the colonizer plants, TOC, and TN. The direction of the arrows for individual factor indicates an increasing concentration of that factor and the length of the arrows indicate the degree of correlation with the represented axes. The numbers correspond to the bacterial families (A) and fungal classes (B) in the keys at right and are ranked according to abundance.
